# Supplementary material for: A set of multi-entry identification keys to African frugivorous flies (Diptera, Tephritidae)
Source: Zookeys. 2014 Jul 24;(428):97–108. doi: 10.3897/zookeys.428.7366 (PMC4143993; doi:10.3897/zookeys.428.7366)
Supplement: Supplementary material 6 — Key to Ceratitis [file zookeys-428-097-s006.zip › SF6_ZooKeys_key to Ceratitis/key/SF6_ZooKeys_key to Ceratitis/Media/Html/Ceratitis mlimaensis.htm]

***Ceratitis*** **(*Ceratalaspis*)
*mlimaensis* De Meyer**

*Ceratitis* (*Ceratalaspis*)
*mlimaensis* De Meyer, 1998 : 281.

Body
length: 4.31 (4.25-4.45) mm; wing length: 4.54 (4.40-4.70) mm.

Male

Head.
Antenna yellow-orange. Third antennal segment two times as long as second
segment. Arista with short hairs over entire length. Frons yellow to orange;
with short scattered hairs which are largely the same colour as the frons; flat
to convex, in lateral view not distinctly projecting forwards at antennal
implant. Chaetotaxy normal for subgenus.

Thorax.
Postpronotum only slightly paler in colour than centre of mesonotum,
yellow-white; unspotted. Ground colour of mesonotum pale with yellow-orange
tinge; presutural median spot absent (in BMNH specimen, invisible in USNM
specimen because of insect pin). Chaetotaxy normal for subgenus. Scapular setae
black. One or two anepisternal bristles, if second present then lower reduced
in size. Scutellum mainly yellow; with three black separate markings apically
except at ventral margin where narrowly joining; basally no distinct spots.
Subscutellum pale with three dark separate spots.

Legs
yellow; setation typical for subgenus, mainly pale especially on femora. Posterior
and posterodorsal rows on fore femur pale.

Wing
bands with markings yellow-brown. Banding, marginal band continuous; cubital
band free; medial band joined with marginal band; crossvein r-m before middle
of discal cell. Crossvein dm-cu posteriorly more inwards than anteriorly.

Abdomen.
Yellow. Setation and banding typical for subgenus.

Female.

As
male except for the following� characters: mesonotum with presutural median
spot present. Crossvein r-m well before middle of discal cell. Oviscape as long
as abdominal terga 3-6 combined.�

(Description
after De Meyer, 2008)
